# Brazilian transcultural adaptation of an instrument on communicative strategies of caregivers of elderly with dementia

**DOI:** 10.1590/1980-57642016dn11-030005

**Published:** 2017

**Authors:** Lais Lopes Delfino, Ricardo Shoiti Komatsu, Caroline Komatsu, Anita Liberalesso Neri, Meire Cachioni

**Affiliations:** 1 Doutoranda em Gerontologia no Programa de Pós-Graduação em Gerontologia, Faculdade de Ciências Médicas, Universidade Estadual de Campinas, Campinas, SP, Brasil.; 2 MD, MSc, PhD, Professor Doutor, Chefe da Disciplina de Geriatria e Gerontologia, Faculdade de Medicina de Marília, Marília, SP, Brasil.; 3 Graduanda (4º ano) em Medicina, Escola de Medicina e Cirurgia, Universidade Federal do Estado do Rio de Janeiro, Rio de Janeiro, RJ, Brasil.; 4 PhD, Professora Titular, Faculdade de Ciências Médicas, Universidade Estadual de Campinas, Campinas, SP, Brasil.; 5 PhD, Professora, Escola de Artes, Ciências e Humanidades, Universidade de São Paulo, São Paulo, SP, Brasil.

**Keywords:** communication, caregiver, dementia, comunicação, cuidador, demência

## Abstract

Communication with patients with dementia may be a difficult task for caregivers.

**OBJECTIVE:**

The aim of this study was to produce a Brazilian transcultural adaptation of an instrument developed in Canada, called the Small Communication Strategies Scale, composed of 10 items constructed from 10 communicative strategies most recurrent in a literature survey.

**METHODS:**

Drawing on understanding of the construction of the Small Communication Strategies Scale (SCSS), a Brazilian Portuguese version of the instrument was devised through the following steps: translation, back-translation and semantic-cultural adaptation by a specialized linguist in English-Portuguese translations and application of the comprehension test for the version produced in a group of caregivers of elderly individuals with dementia.

**RESULTS:**

The transcultural equivalence process was performed and two items of the SCSS needed adapting to the Brazilian context. After changes suggested by a specialized linguist, the final version was applied to 34 caregivers and the transcultural equivalence considered satisfactory.

**CONCLUSION:**

The Brazilian version of the instrument was successfully transculturally adapted for future validation and application in Brazil.

## RESUMO

A comunicação com pacientes com demência pode ser muito difícil para os cuidadores.

**OBJETIVO:**

Este estudo objetivou obter a adaptação transcultural de um instrumento desenvolvido no Canadá, denominado Small Communication Strategies Scale, composto por 10 itens que foi construído a partir de 10 estratégias comunicativas que mais apareciam em um levantamento bibliográfico.

**MÉTODOS:**

A partir da compreensão da construção da *Small Communication Strategies Scale* (SCSS), realizou-se uma versão em português brasileiro desse instrumento, por meio das seguintes etapas: tradução, retrotradução e adequação semântico-cultural realizada por uma linguista especialista em versões inglês-português e o teste da compreensibilidade da versão obtida num grupo de cuidadores de idosos com demência.

**RESULTADOS:**

A equivalência transcultural foi realizada, e no teste de compreensibilidade apenas dois itens do SCSS precisaram ser adaptados para o contexto brasileiro: 2) lentamente e devagar – optou-se por *devagar*; 6) concentrar a atenção e *prestar atenção* – adotou-se prestar atenção, neste mesmo item, entre os termos: estratégias de reparação na conversa e fazer interferências quando necessário for, optou-se por *fazer interferências quando necessário for*.

**CONCLUSÃO:**

A versão brasileira do instrumento foi adaptada transculturalmente com sucesso e está pronta para ser validada e aplicada no Brasil.

## INTRODUCTION

People with dementia suffer a variety of language difficulties during the disease process that affect communication. For example, in the mild stages of Alzheimer's disease (AD), individuals often have difficulty naming objects.^1^ The ability to read, write and comprehend words and sentences remain relatively intact compared to healthy elderly individuals. In the moderate stages, difficulties with the use of functional language, concept formation, comprehension and writing become increasingly more relevant. Subjects fail to understand questions and sentences, making conversation difficult.^2^ At advanced stages, the elderly can communicate in an unintelligible way or become totally mute.^3^ In some cases, non-verbal communication, such as body gestures, allow word recognition to be maintained in advanced AD, minimally enabling the communicative elements to be interpreted.^4^

Therefore, with dementia progression, communication difficulties become more considerable. In the study by Rosa et al.,^5^ who identified the needs of caregivers, it was shown that 83% needed information on how to communicate effectively with people with dementia.

The communicative strategies used by caregivers influence the way the elderly with AD respond to the situation. In a systematic review conducted by Delfino and Cachioni,^6^ the communicative strategies identified in the studies investigated to reduce dysfunctional behaviors were to avoid reality-oriented techniques, to provide verbal and non-verbal support, and to use the solution-oriented approach rather than confront the elderly. On the other hand, the communicative style that contributed to the agitation of the elderly with dementia was the use of language in an inappropriate way, whereby the elderly were unable to understand infantile and controlling communication.

Research suggests that both the dysfunctional responsiveness and the communication problem may be tied to tone (including vocal quality, intonation and loudness) or the communicative style of the interlocutor.^7^ In particular, investigations have shown that people with dementia respond negatively to a negative communicative tone.^8^ The use of critical emotional expression or roughness was found to be predictive of increased negative behaviors^9^ and reduced responsiveness in conversation.^10^ In contrast, caregivers who exhibited patience and smoothed tone were able to facilitate responsiveness^10^ and reduce dysfunctional behavior.^11^ Therefore, the relationship between communicative strategies used by caregivers and behavioral problems in the elderly with dementia seems clear. Problems that caregivers face may include lack of awareness of the symptoms of dementia, specific changes in communication with people with dementia, and methods for achieving good communication. This lack of knowledge about challenging behavior and its possible causes often leads to failure of communication, fear, and misunderstanding.^12^ Communication difficulties have been associated with relationship conflicts, social isolation, depression, burden and stress for caregivers.^7^^,^ 13

Family caregivers should be aware when a set of strategies work well so they can replicate it under other circumstances. Caregivers should also recognize the unstable profile of dementia, which means that communicative strategies that work in the initial phase may not work in the final phase.^14^ Thus, investigating which communicative strategies are used by caregivers and which facilitate interactions between elderly and caregivers are important for managing behaviors and daily life of elderly with dementia.

In a systematic review of communicative strategies used by caregivers, it has been shown that there are few scales and instruments that quantitatively evaluate this issue. The heterogeneity of the methodological approaches used to observe patterns of interaction between caregiver and elderly with AD was observed in the studies investigated. Filming, recording, focus groups, open interviews and coded observation were the main techniques used. By contrast, only two scales were identified in these studies.^6^ One of these instruments was the scale chosen for cross-cultural adaptation into Portuguese.

The Small Communication Strategies Scale, developed by specialists in Canada, is a 10-item questionnaire that was constructed from the 10 communicative strategies most recurrent in the literature for AD caregivers. The selection of the strategies was based on whether they fulfilled all of the following criteria: [A] the strategy recurred across publications; [B] the strategy involved modified language and behavior or environmental features as ways of accommodating the communication needs of the person with AD; and [C] the strategy would likely be understood by lay caregivers.^16^ The SCSS was chosen as a gold standard for the criteria adopted in its construction. The scale items include behaviors (1, 2, 7, 8, 9, 10), non-verbal communication (2, 6), and simplified communication (3, 4, 5), elements that are consistent with the communication needs of persons with AD.

The aim of the present study was to carry out transcultural adaptation of the Small Communication Strategies Scale, since no instruments for investigating communicative strategies used by caregivers of elderly with AD were found in the Brazilian literature.

## METHODS

The translation of the instrument that includes the Small Communication Strategies Scale questionnaires was authorized by its authors and this project was approved by the Ethics Committee of the Faculdade de Ciências Médicas, Universidade Estadual de Campinas (CAAE: 49137515.4.0000.5404). It should be noted that all participants were aware of the study and signed the Free and Informed Consent Form, as determined by Resolution No. 466/2012 of the National Health Council (Brazil).

### Instrument.

A 10-item questionnaire that listed the 10 strategies yielded by a literature search was produced. A search of the literature (paper and electronic) was conducted to identify communication strategies recommended for family AD caregivers. The authors of the scale selected a representative sample of widely available sources. Two major sources were selected: [A] national associations that provide information and/or assistance to family caregivers (e.g., the Alzheimer's Association [United States], the Alzheimer Society of Canada); and [B] authored books intended for the AD family caregiver.^15^^-^ 19 Besides each strategy there were two categories of responses requested from the caregiver. First, the caregiver was asked to indicate how often they used each strategy when communicating with the person with AD by checking one of five options: always, frequently, occasionally, rarely, or never. Second, they had to indicate how much they feel the strategy improves communication by checking one of four options: very much, quite a bit, somewhat, or not at all. The authors of the scale consider that although it is likely that the frequency of strategy use depends on the occasions in which a particular strategy can be used appropriately, they argued that all the strategies can be used appropriately in every communicative interaction at home. Thus, it was assumed that caregivers are responding to the question "frequency of use" with how often they implement the strategy in any given communicative interaction. We found no cross-cultural adaptation and validation in other languages and authors did not report test reliability results in the studies using SCSS.^10^^,^ 15

### Procedures.

**Figure 1:**
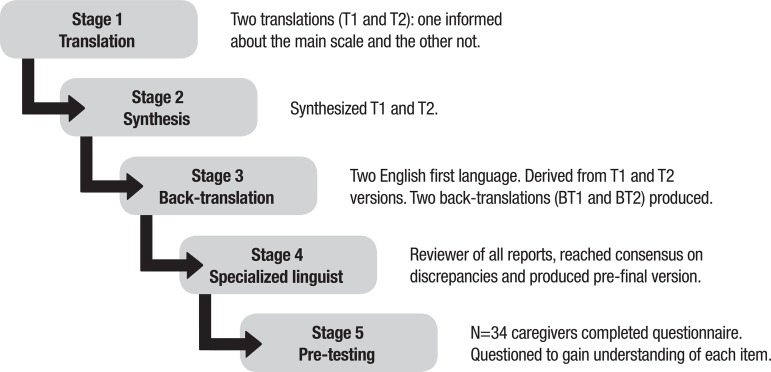
Graphic representing stages of the cross-cultural adaptation.

Drawing on the understanding of the construction of the Small Communication Strategies Scale (SCSS), a Brazilian Portuguese version of the instrument was devised, following the guidelines proposed by Beaton et al.^20^ based on an improved method involving other procedures and techniques widely used for cultural adaptation of health measures and other fields of knowledge. In this study, 5 steps were performed (Figure 1).

In the first stage, the scale was translated into the Portuguese language of Brazil; two translations were carried out by independent translators who did not exchange information with each other. In the second stage, synthesis of the first version in Brazilian Portuguese was performed. Using the scale and the two translations, a third Brazilian professional, a Portuguese-English bilingual gerontologist, performed the synthesis of the two translations. A detailed report was produced describing the discrepancies that occurred and the reasons for the choices made. Using the synthesized version in Portuguese, the third stage entailed producing the back-translations. Versions were prepared by two back-translators, whose mother tongue was English, the same language as the original scale, without access to the published version of the scale. The back-translators were British, residing in Brazil for more than ten years.

The fourth stage involved consolidation of the semantically acceptable version for Brazilian Portuguese. This step was carried out by a committee of experts who reviews all translations and reached a consensus on any discrepancies. Employing all versions of the scale, a specialized linguist in English-Portuguese translations prepared a report to produce the version with adapted semantic equivalence, after the approval of all those involved.

The last stage consists of testing the validity and quality of the content, generally applied in a group of 30 to 40 people.^20^ Thus, after completing the scale, 34 subjects were interviewed and questioned about the meaning of each item of the scale and their respective chosen answer. This process ensures that the version has applicability equivalence.

### Subjects.

The final culturally-adapted version for Brazilian Portuguese was submitted to evaluation by a convenience sample of 34 caregivers. Only caregivers who provided day-to-day care for at least four hours a day and caring for elderly people who had been diagnosed with Alzheimer's disease participated in the study, according to the criteria recommended by the International Working Group (IWG) and The US National Institute of the Aging-Alzheimer's Association.^21^ Caregivers caring for elderly patients with terminal comorbidity and with a life expectancy of less than 6 months, according to the medical evaluation, were excluded. All caregivers co-resided with the elderly with AD and most (44.1%) did not present depressive symptoms (Table 1). In a single and individual interview, professionals were invited to read and comment on each item in the scale.

**Table 1:** Sociodemographic characteristics of caregivers of patients with Alzheimer's disease.

		Frequency	Percentage
Gender	Male	8	16
	Female	26	84
Age (years)	< 50	5	15
	51 to 60	18	53
	> 60	11	42
Education (years)	< 4	2	6
	5 to 11	5	15
	> 12	27	79
Professional caregiver	Yes	22	65
	No	12	35
Income	1.5 to 3 SM	3	8,8
	3.5 to 5 SM	12	35,3
	> 5 SM	19	55,9
Depressive Symptoms (BDI scores)	0-15	15	44,1
	16-20	13	38,2
	21-29	6	17,6
Hours spent caring	5 to 10 hours	19	56
	>11 hours	15	44

BDI: Beck's Depression Inventory.

## RESULTS

The data obtained are given in Table 2, which contains the steps of the cross-cultural adaptation of the Small Communication Strategies Scale (SCSS) to obtain an equivalent Brazilian version of the *Escala Breve de Comunicação de Estratégias de Comunicação* (EBEC). Within this framework, the original items of the SCSS can be observed; the synthesis of the translations comprising the first Brazilian Portuguese version; the two back-translations in full; the consolidation of a semantically acceptable version and the EBEC, which corresponds to the final version culturally adapted to Brazilian Portuguese.

**Table 2:** Results of the semantic-cultural adaptation process of the Small Communication Strategies Scale (SCSS) for Brazilian Portuguese. Campinas-SP, 2017.

Question	Original version	Synthesis of the translations	Back-translation 1	Back-translation 2	Consolidation of a semantically acceptable version	Brazilian cross-culturally adapted version
1	Eliminate distractions	Eliminar distrações	Eliminating distractions	Eliminate distractions	Eliminar distrações	Eliminar distrações
2	Approach your spouse slowly and from the front, establish and maintain eye contact	Aproximar-se do seu cônjuge lentamente e pela frente, estabelecendo e mantendo contato visual.	Approaching your spouse slowly and from the front, establishing and maintaining eye contact	Slowly approach your spouse from the front, while establishing and maintaining eye contact.	Aproximar-se do seu cônjuge lentamente pela frente, estabelecendo e mantendo contato visual.	Aproximar-se do seu cônjuge devagar e de frente, estabelecendo e mantendo contato visual.
3	Use one-idea sentences	Usar frases simples	Using simple sentences	Use simple phrases	Usar frases simples	Usar frases simples
4	Speak at a normal rate and without exaggerated intonation	Falar de maneira normal e sem alterar o tom de voz.	Speaking normally, without changing your tone of voice	Speak naturally without changing the tone of voice.	Falar em ritmo normal e sem alterações exageradas no tom de voz.	Falar em ritmo normal e sem alterações exageradas no tom de voz.
5	Ask questions that do not place demands on recent memory	Fazer perguntas que não exijam o uso da memória recente	Asking questions that don't require the use of short-term memory	Ask questions that do not require using recent memory	Fazer perguntas que não exijam muito da memória recente.	Fazer perguntas que não exijam muito da memória recente.
6	Use focused management and repair strategies in conversation	Utilizar estratégias de manejo e recuperação do foco durante a conversa	Using strategies for managing and regaining focus during the conversation	Use strategies for handling and regaining focus during the conversation	Concentrar a atenção em estratégias de condução e reparação na conversa.	Prestar atenção na condução da conversa e fazer interferências apropriadas quando necessário for.
7	Invite spouse's participation in the conversation	Solicitar a participação do cônjuge na conversa.	Requesting your spouse's participation in the conversation	Ask your spouse to participate in the conversation	Convidar o cônjuge a participar da conversa.	Convidar o cônjuge a participar da conversa.
8	Encourage and facilitate spouse's sustained participation in the conversation	Incentivar e facilitar a participação contínua do cônjuge na conversa.	Encouraging and facilitating your spouse's participation in the conversation	Encourage and facilitate your spouse's continuous participation in the conversation	Incentivar e facilitar a participação contínua do cônjuge na conversa.	Incentivar e facilitar a participação contínua do cônjuge na conversa.
9	Orchestrate and partner with spouse during conversation	Conciliar e ser parceiro (a) do seu cônjuge durante a conversa	Reconciling with and being on the same team as your spouse during the conversation	Accommodate and be your spouse's partner during the conversation	Conciliar e atuar como parceiro do cônjuge durante a conversa.	Conciliar e atuar como parceiro do cônjuge durante a conversa.
10	Honor and respect spouse's perspective and needs	Honrar e respeitar as expectativas e as necessidades do cônjuge	Honoring and respecting your spouse's expectations and needs	Honour and respect your spouse's expectations and needs	Honrar e respeitar os pontos de vista e as necessidades do cônjuge.	Honrar e respeitar os pontos de vista e as necessidades do cônjuge.

Items 2, 4, 5, 6, 7, 9 exhibited differences in written form by the two independent back-translators. However, after analyzing them together, it was concluded that there was no semantic divergence, that is, the terms were equivalent. Based on the suggestions obtained from the linguist specialized in English-Portuguese versions and the caregivers interviewed, the version underwent changes for its cultural refinement. The items that presented differences were: 2) *lentamente* and *devagar - devagar* was chosen; 6) *concentrar a atenção* and *prestar atenção - -*prestar atenção was adopted, on this same item, difference between the terms: *estratégias de reparação na conversa* and *fazer interferências quando necessário for*, where *fazer interferências quando necessário for* was chosen.

In relation to the question on frequency of use of the strategy (section 1), after the semantic equivalence steps, responses to the following were considered: *Com que frequência você utiliza a estratégia ao se comunicar com seu cônjuge*? (How often do you use the strategy when communicating with your spouse?). On the five-point Likert scale, scores range from 10 to 50 points. Low scores indicate that caregivers use communicative strategies more frequently. Regarding the question that seeks to investigate the caregiver's self-assessment of the effectiveness of each strategy (section 2), this was defined as: *O quanto você acredita que a estratégia seja capaz de melhorar a comunicação com seu cônjuge*? (How much do you feel the strategy improves communication with your spouse?). In this section, scores range from 10 to 40, and low scores indicate that caregivers consider that strategies improve communication with their spouses.

Family caregivers considered the EBEC an important communication scale for self-assessment of the way they communicate with their family members. They considered that to answer the scale it is important to identify the quality of the relationship between the patient with AD and their spouse before the onset of illness, since it can contribute to an understanding of the use of the communicative strategies investigated. They considered it a simple and quick scale to answer.

## DISCUSSION

The literature documents numerous studies on the communication deficits that the elderly with dementia present, using different methodologies and a diverse variety of instruments that aim to investigate this cognitive area. However, there are few studies investigating the communicative strategies used by caregivers of the elderly.^6^ Thus, the present study represents an effort to advance in this area of research by translating and adapting a questionnaire on communicative strategies used by caregivers of elderly people with dementia into Portuguese.

The cross-cultural adaptation evaluated and reached the equivalences of the SCSS in the fields: semantic and idiomatic, which ensured the same meaning of the words and use of expressions; conceptual, which verified the theoretical construct; cultural, which checked the situations presented in the scale; and criterion, which investigated the normative interpretation of the items of the scale studied.

We chose the simplest terms in the transcultural translation and adaptation process so that caregivers could readily understand each question. Although the education level of caregivers answering the EBEC was relatively high, care was taken to observe whether those caregivers with low education level also understood what was being asked.

It is important to note that elements of communication may not be explored in scales containing closed-ended questions. It is known that non-verbal communication is a relevant form of expression that qualifies human interaction, imparting feelings, emotions, qualities and a context that allows the individual not only to understand what words mean, but also to understand the feelings of the interlocutor.^22^ However, the scale selected in this study may support a qualitative investigation of the communicative interaction between caregiver and patient.

The use of this scale can identify whether caregivers use these communicative strategies and whether they can contribute toward improving communicative standards. Such data are important for the health team to help caregivers improve the relationship between caregiver and the elderly with AD, given the positive effects reported by the literature when communication is effective.

When evaluating the results of this study the small sample size should be taken into consideration. Future studies with a larger number of participants are necessary to confirm these results. Results showed that the Portuguese version of the SCSS can be a good empirical research tool for elderly caregivers. The stages of translations and back-translations indicated that the Portuguese version is linguistically true to the original questionnaire, and its adequacy was also confirmed by the expert linguist and the interviewed caregivers.

The SCSS adaptation is expected to stimulate further research investigating the communicative strategies used by caregivers. Future research should continue to investigate such strategies in different processes and dementia stages.
